# Salidroside Selectively Binds to SEC23A and Ameliorates Psychological Stress-Induced Hyperpigmentation

**DOI:** 10.3390/ph19030487

**Published:** 2026-03-16

**Authors:** Man Yang, Xiaoyu Sun, Da Wang, Huizhong Nie, Kang Cheng, Jie Gu, Lu Chen, Yuxuan Zhang, Lingli Yang, Ichiro Katayama, Yiming Li, Huali Wu

**Affiliations:** 1 Department of TCM Chemistry, School of Pharmacy, Shanghai University of Traditional Chinese Medicine, Shanghai 201203, China; 2 Shanghai Inoherb Cosmetics Co., Ltd., Shanghai 200080, China; 3 Development and Planning Department, Shanghai University of Traditional Chinese Medicine, Shanghai 201203, China; 4 Pharmaceutical Research Laboratory, Yueyang Hospital of Integrated Traditional Chinese and Western Medicine, Shanghai 200437, China; 5 Department of Pigmentation Research and Therapeutics, Graduate School of Medicine, Osaka Metropolitan University, Osaka 530-0001, Japan

**Keywords:** SAL, psychological stress, melanin, SEC23A, hyperpigmentation

## Abstract

**Background/Objectives:** Psychological stress triggers excessive melanin deposition via neuroendocrine pathways, yet targeted interventions for stress-induced hyperpigmentation remain limited. Salidroside (SAL) exhibits established depigmenting effects in UV-induced models and possesses neuroprotective properties. This study investigated SAL’s efficacy in psychological stress-induced hyperpigmentation and elucidated its underlying mechanisms. **Methods:** B16F10 melanocytes, C57BL/6J mice, zebrafish, and human foreskin organ cultures were subjected to stress factor (Substance P/cortisol) or α-MSH/IBMX stimulation to model psychological stress-induced and canonical cAMP-driven hyperpigmentation, respectively. Melanin content, tyrosinase activity, melanosome maturation (transmission electron microscopy/HMB45 staining), and melanogenic protein/mRNA expression were assessed. Drug Affinity Responsive Target Stability (DARTS) assays, molecular docking, and SEC23A siRNA knockdown were employed to identify and validate SAL’s molecular target and downstream signaling pathways. **Results:** SAL dose-dependently reduced melanin content, tyrosinase activity, and TYR/TRP-1/DCT expression in SP/Cort-stimulated melanocytes, exhibiting greater potency (200 μM) than in IBMX-induced models (400 μM). SAL reversed SP/Cort-induced hyperpigmentation in human skin explants, zebrafish, and C57BL/6J mice, and normalized melanosome number/maturation. DARTS and molecular docking identified SEC23A as a direct SAL-binding target. SP/Cort specifically upregulated SEC23A, which SAL suppressed. SAL concurrently activated the SEC23A-p-ERK-MITF axis and inhibited the NK1R-p38-MITF axis in the stress model. SEC23A knockdown potentiated SAL’s anti-melanogenic effects specifically in SP/Cort-stimulated cells. Conversely, in IBMX-induced models, SEC23A remained unchanged, and SAL acted via PKA/CREB, PI3K/AKT, and Wnt/β-catenin pathways. **Conclusions:** SEC23A is a novel core target in psychological stress-induced hyperpigmentation. SAL selectively binds SEC23A to inhibit stress-induced melanogenesis via dual ERK and p38 MAPK signaling axes, demonstrating etiological specificity distinct from canonical cAMP pathway inhibition.

## 1. Introduction

The degree and distribution of skin pigmentation are pivotal for sustaining skin physiological functions. Beyond precisely regulating skin phenotype, they also serve an indispensable barrier function in protecting against ultraviolet (UV)-induced oxidative stress and DNA damage [1]. A multitude of exogenous factors are well-documented to perturb melanin metabolic homeostasis and induce aberrant upregulation of melanogenesis, including ultraviolet radiation (via direct activation of melanocyte PKA-cAMP signaling pathways), pro-inflammatory mediators (via modulation of the inflammatory microenvironment), and psychological stress [2,3,4,5]. Unlike “direct-acting factors” such as UV and inflammation, psychological stress influences melanin synthesis indirectly through the cutaneous neuroendocrine axis, involving more complex signaling pathways. However, clinical interventions specifically targeting this type of stress-induced pigmentation remain limited.

Human skin displays a high degree of anatomical and physiological integration with the nervous and endocrine systems, which collectively constitute a sophisticated functional network termed the cutaneous neuroendocrine regulatory axis. This network is the core conduit through which psychological stress affects skin function [6]. As the primary barrier interfacing with both the internal and external environments, the skin can sense stress stimuli and elicit adaptive responses through autocrine/paracrine pathways or specific signaling molecules (i.e., hormones, neurosecretory factors, neurotransmitters) [7]. Under stress, the central hypothalamic–pituitary–adrenal (HPA) axis is activated in a cascade, sequentially releasing corticotropin-releasing hormone (CRH), adrenocorticotropic hormone (ACTH), and cortisol (Cort), generating systemic neuroendocrine signals [8,9]. Concurrently, a local HPA axis (first proposed in 1996) and the pro-opiomelanocortin (POMC) system exist within the skin itself, capable of responding to stress by locally synthesizing CRH, ACTH, and neuropeptides such as Substance P (SP) and Calcitonin Gene-Related Peptide (CGRP) [10,11,12]. Existing evidence confirms the existence of a “stress-pigmentation axis”. Firstly, clinical data indicate that 30.2% of dermatology consultations are related to psychosomatic skin diseases, and the prevalence of depression/anxiety is significantly higher in patients with pigmentation disorders like melasma and ephelides (freckles) [13,14]. Then, animal studies also show that chronic restraint stress (CRS) can upregulate the density of SP-positive nerve fibers in mouse skin, and stress-induced increases in serum ACTH/Cort can directly regulate melanogenesis [15,16,17,18]. However, critical knowledge gaps persist: although SP and Cort are recognized as core upstream molecules in stress-mediated pigmentation, their downstream core targets regulating melanogenesis remain unidentified. Furthermore, the regulatory mechanisms governing the processing of melanogenesis (such as melanosome maturation and transport) under stress conditions require deeper investigation.

Salidroside (SAL), the characteristic active compound of *Rhodiola rosea* L., has been demonstrated to possess multiple biological functions, including roles in anti-tumor, anti-inflammatory, and cardioprotective activities, as well as established neuroprotective effects potentially intervening in neuropsychiatric disorders like anxiety and depression [19,20]. In the field of pigmentation, preliminary studies indicated that *Rhodiola rosea* L. aqueous-alcohol extracts can inhibit melanogenesis via the CREB/MITF/tyrosinase pathway [21]. Subsequent mechanistic research further revealed that in UVB-induced pigmentation models, SAL can target P4HB protein, regulate the assembly of the IRF1/USF1 transcriptional complex, and concurrently suppress both skin inflammation and aberrant melanogenesis [22]. Consequently, the anti-pigmentation effects of SAL have only been validated in “UVB-induced” models. Key questions regarding its efficacy against “psychologically stress-induced pigmentation,” potential mechanistic differences from UVB models, and its ability to target core pathways of stress-mediated melanogenesis remain unanswered, limiting SAL’s potential application value in stress-related pigmentation disorders. Our team’s previous work has verified that SP can significantly aggravate pigmentation via signal crosstalk with Cort [23]. This finding indicates that the SP/Cort co-induction model effectively mimics the psychological stress state, thereby offering an ideal experimental model for exploring the role of SAL in this context.

Based on this foundation, the present study adopted a multi-factor pigmentation model and integrated multiple techniques to perform the following work: First, we established an SP/Cort-induced B16F10 melanocyte pigmentation model (to simulate psychological stress) and a classic α-MSH/IBMX-induced pigmentation model (as a control). By measuring melanin content, tyrosinase activity, and mRNA/protein expression of TYR, TRP-1, and DCT, we evaluated SAL’s inhibitory effect on neuroendocrinally-mediated aberrant pigmentation. Second, using transmission electron microscopy (TEM) to observe melanosome number and maturation stage, combined with immunofluorescence (HMB45 staining) to analyze melanosome distribution and maturity, we aimed to clarify SAL’s impact on melanosome development and transport. Third, utilizing Drug Affinity Responsive Target Stability (DARTS) assays and molecular docking screening, we jointly validated SAL’s potential binding target SEC23A. Combined with siRNA-mediated target *Sec23a* knockdown experiments, we dissected the downstream molecular mechanism by which SAL regulates stress-induced pigmentation. Finally, in C57BL/6J mice (back skin pigmentation model), zebrafish (whole-body pigmentation model), and human foreskin organ culture systems, we validated the in vivo and ex vivo anti-pigmentation activity of SAL and compared its effects in the stress model versus the common model.

To establish an in vitro model of psychological stress-induced hyperpigmentation, B16F10 cells were co-stimulated with Substance P (SP; 10 nM) and cortisol (Cort; 10 µM). The concentrations of SP and Cort were selected based on previous studies that reported elevated levels of these mediators in the skin or serum under chronic stress conditions. Specifically, SP at nanomolar ranges has been shown to activate its receptor NK1R and induce melanogenic responses in melanocytes, Similarly, cortisol at micromolar concentrations has been demonstrated to mimic the local tissue levels observed in chronic stress and to directly influence melanocyte function [24,25,26]. This SP/Cort co-stimulation model thus aims to recapitulate the core neuroendocrine component of the cutaneous stress response.

## 2. Results

### 2.1. Under SP/Cort Exposure, SAL Exhibits Greater Efficacy on Alleviating Hyperpigmentation Effects

**Figure 1:**
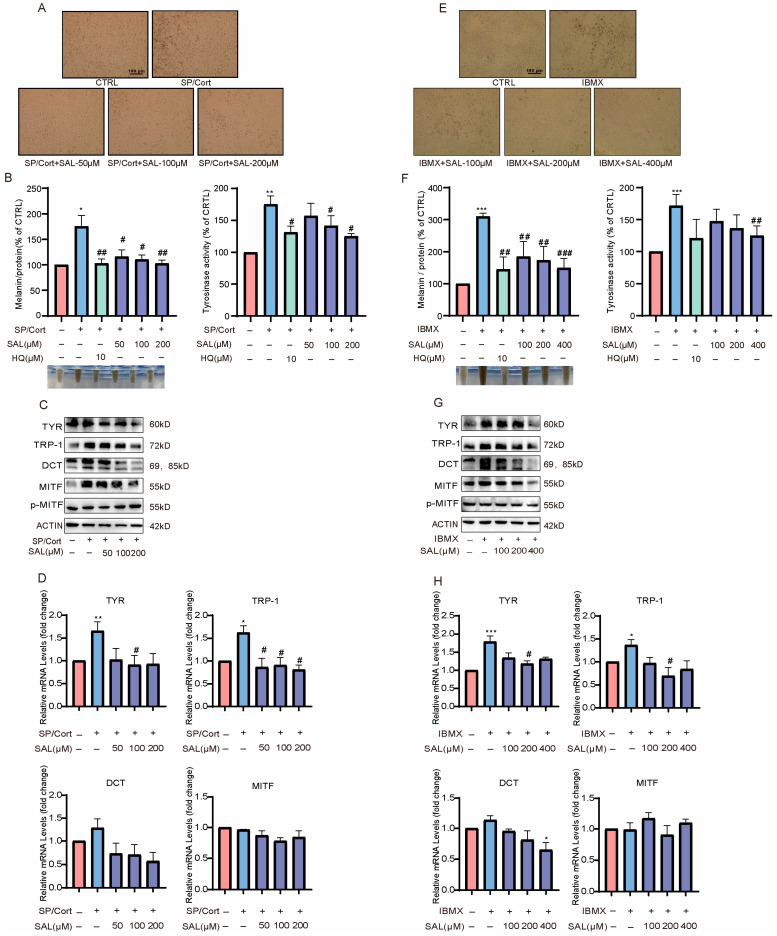
SAL alleviates hyperpigmentation more effectively in SP/Cort-induced than in IBMX-induced B16F10 cells. (**A**–**D**) SP/Cort (10 nM/10 μM) model: (**A**) Representative images of intracellular melanin granules. (**B**) Effects of SAL (50, 100, 200 μM) or hydroquinone (HQ, 10 μM) on melanin content and tyrosinase activity. (**C**) Western blot analysis of MITF, p-MITF, TYR, TRP-1, and DCT. (**D**) mRNA expression levels of *Mitf, Tyr*, *Trp-1*, and *Dct* measured by RT-qPCR. (E–H) IBMX (100 μM) model: (**E**) Representative images of melanin granules. (**F**) Melanin content and tyrosinase activity after SAL (50, 100, 200 μM) or HQ treatment. (**G**) Protein expression of melanogenic enzymes. (**H**) mRNA levels of melanogenic genes. Data are presented as mean ± SEM (*n* = 3 per group) * *p* < 0.05, ** *p* < 0.01, *** *p* < 0.001 compared with the control group (SP/Cort− or IBMX−, SAL−) # *p* < 0.05, ## *p* < 0.01, and ### *p* < 0.001 vs. (SP/Cort or IBMX) are considered to be significant. Representative images from three independent experiments is are shown.

The effect of SAL on stress-induced hyperpigmentation was evaluated in B16F10 cells. Microscopic examination revealed a marked accumulation of melanin granules in both the SP/Cort and IBMX(3-isobutyl-1-methylxanthine) groups (Figure 1A,E). SAL treatment reduced pigmentation in a concentration-dependent manner (Figure 1A,E), which was further supported by visual assessment of precipitate color in SAL-treated cells (Figure 1B,F).

To ensure that the anti-melanogenic effects of SAL were not attributable to cellular toxicity, we assessed its impact on B16F10 cell viability under identical treatment conditions. As shown in Supplementary Figure S1 SAL at concentrations up to 400 μM did not induce any significant reduction in cell viability after 72 h of treatment. These results confirm that the observed reductions in melanin synthesis and tyrosinase activity are due to specific biochemical inhibition rather than nonspecific cytotoxic effects.

Since tyrosinase (TYR) is the key rate-limiting enzyme in melanin biosynthesis, its activity was quantitatively assessed using the L-DOPA oxidation assay. The results demonstrated that SAL dose-dependently suppressed the upregulation of TYR activity in both the SP/Cort and IBMX-induced models (Figure 1B,F). Notably, under SP/Cort-induced stress, SAL significantly inhibited melanin synthesis and tyrosinase activity at a concentration of 200 μM. In contrast, a higher concentration of 400 μM was required to elicit significant effects in the IBMX-induced model (Figure 1B,F). In addition, the positive control hydroquinone (HQ, 10 μM) exhibited a stronger inhibitory effect on melanin content and tyrosinase activity than SAL in both models (Figure 1B,F). This is consistent with the known potent depigmenting activity of HQ. However, HQ is associated with significant safety concerns, including cytotoxicity, melanocyte toxicity, and exogenous ochronosis with long-term use [27,28].

These findings indicate that SAL exhibits stronger anti-pigmentation efficacy in the SP/Cort-challenged model compared to the conventional IBMX-induced model.

The Western blot experiment was carried out to explore the alterations of key proteins regulating melanin synthesis by SAL. The results showed that both SP/Cort and IBMX stimulators upregulated the gene and protein expression of TYR and tyrosinase-related proteins (TRP-1 and DCT), which were significantly reversed by SAL treatment (Figure 1C,D,G,H, Supplementary Figure S2A,B). MITF plays a pivotal role in the transcriptional regulation of TYR protein. As showed, the protein expression levels of MITF were significantly upregulated by SP/Cort and IBMX stimulation, which could be reversed by SAL intervention (Figure 1C,G, Supplementary Figure S2A,B). Still, p-MITF protein level and MITF mRNA level both showed no alteration (Figure 1C,D,E,G, Supplementary Figure S2A,B). This outcome implies that SAL inhibits pigment synthesis through downregulating the expression of MITF, TYR, and tyrosinase-related proteins and the activity of tyrosinase.

### 2.2. SAL Reverses Stressful Factor SP/Cort-Induced Hyperpigmentation on the Human Skin-Organ Culture

**Figure 2:**
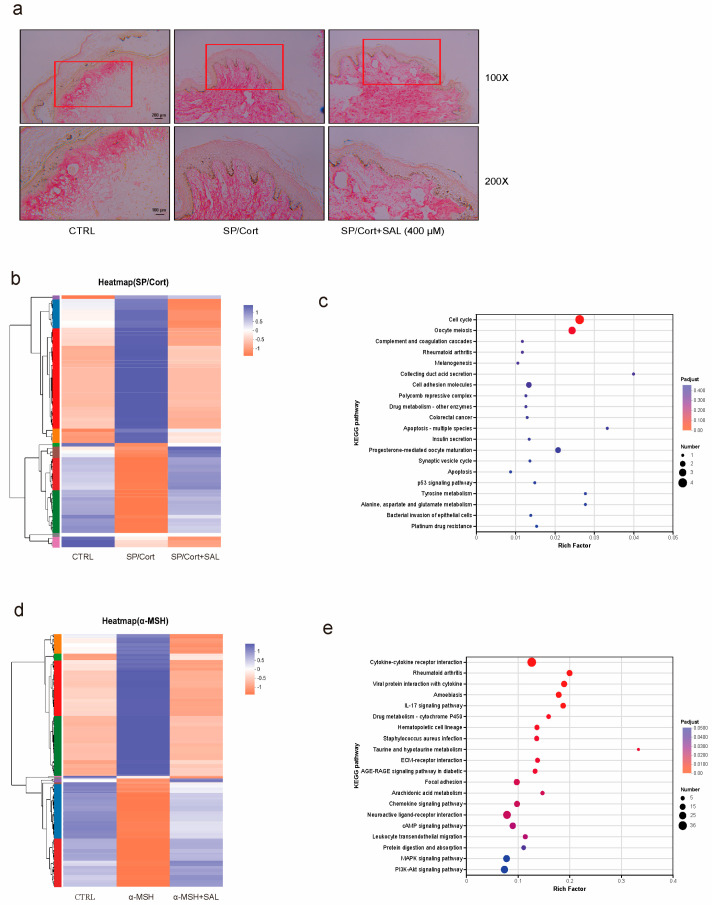
SAL reverses stressful factor SP/Cort-induced hyperpigmentation in human skin organ culture. (**a**) Representative images of healthy human skin tissue stained with Masson-Fontana in the SP/Cort-induced hyperpigmentation model. Images are shown at 100× (scale bar: 200 μm) and 200× (scale bar: 100 μm) magnification. The red squares in the 100× images indicate the regions of interest (ROIs) selected for higher-magnification (200×) imaging and subsequent melanin quantification. (**b**,**d**) Heatmaps of differentially expressed genes (DEGs) among CTRL, SP/Cort or α-MSH, and SAL groups. The color scale represents relative gene expression levels (blue = upregulated, red = downregulated). (**c**,**e**) KEGG enrichment analysis of DEGs: the color gradient represents the adjusted *p*-value (Padjust; red = more significant enrichment) and the dot size represents the number of genes enriched in each pathway.

To further define the inhibitory impact of SAL on pigmentation, we tried to incubate normal human skin tissues with SAL (400 μM) for a week. As described by Masson-Fontana staining, SP/Cort significantly increased skin pigmentation at the dermal–epidermal junction in normal human skin tissue, while SAL (400 μM) could reverse this cutaneous hyperpigmentary phenotype (Figure 2A). Histological observations collectively indicate that SAL attenuates melanogenesis within melanocytes; additionally, the reduced pigment deposition at the dermal–epidermal junction—where melanosomes are typically transferred to keratinocytes—suggests a potential role in modulating melanosomes trafficking to recipient keratinocytes.

To gain deeper insights into the molecular mechanisms of SAL in SP/Cort-induced hyperpigmentation, we conducted a genome-wide transcriptome analysis using RNA-seq. This analysis aimed to identify and characterize the differentially expressed genes (DEGs) induced by SAL in SP/Cort exposure. The cluster heat map was generated to illustrate the significant impact of SAL on SP/Cort and α-MSH-induced DEGs. As the primary inducer of the UVB-responsive signaling cascade (cAMP pathway) in melanocytes, α-MSH triggers this pathway activation, whereas IBMX acts as a cAMP inducer amplifying this signaling downstream. Consequently, our experimental paradigm used α-MSH or IBMX to simulate UVB-initiated cAMP signaling activation, collectively modeling the UVB response in primary human melanocytes and skin tissues. 

In the SP/Cort model, the analysis revealed that a total of 1138 DEGs (374 upregulated and 764 downregulated genes) exhibited significant changes compared to the control and were further influenced by SAL (140 upregulated and 196 downregulated genes) (Figure 2B). According to KEGG analysis, SAL treatment resulted in a significant enrichment of genes involved in melanogenesis (Figure 2C). In contrast, compared to the control, the analysis revealed a total of 680 DEGs that exhibited significant changes in expression levels due to α-MSH treatment (339 upregulated and 341 downregulated genes) and were further influenced by SAL (160 upregulated genes and 374 downregulated genes) (Figure 2D). According to KEGG analysis, SAL treatment significantly enriched genes involved in signaling pathways regulating melanogenesis, such as PI3K/AKT, MAPK, and cAMP signaling pathways (Figure 2E). Collectively, these results offer pivotal clues for elucidating the molecular mechanisms detailed in the following sections.

### 2.3. SAL Reduces Melanosome Number and Inhibits Melanosome Maturation in SP/Cort-Induced Hyperpigmentation Model

**Figure 3:**
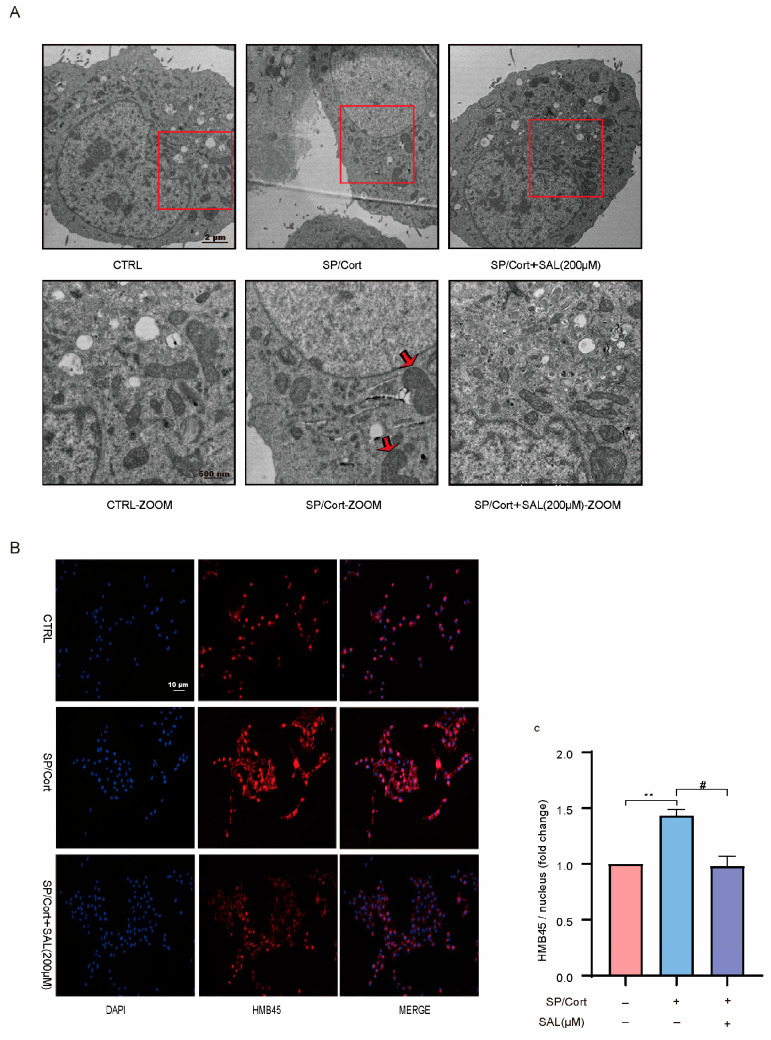
SAL reduces melanosome number and inhibits melanosome maturation in B16F10 cells under SP/Cort-induced hyperpigmentation. (**A**) Representative transmission electron microscopy (TEM) images of melanosomes in B16F10 cells. Upper panels: original images (scale bar: 2 μm); lower panels: zoomed views (scale bar: 500 nm). Red squares in the upper panels indicate regions of interest (ROIs) selected for zoomed imaging. Red arrows in the zoomed panels indicate mature melanosomes. (**B**) Representative immunofluorescence staining images of B16F10 cells (scale bar: 10 μm). Red fluorescence (HMB45, melanoma gp100) labels melanosomes, and blue fluorescence (DAPI) labels cell nuclei. (**C**) Quantification of HMB45 fluorescence intensity (normalized to nucleus). Data are presented as mean ± standard error of the mean (SEM) (*n* = 3 per group). Statistical significance was determined by one-way analysis of variance (ANOVA) followed by Tukey's post hoc test, ** *p* < 0.01 compared with the CTRL group; # *p* < 0.05, compared with the SP/Cort group. Representative images from three independent experiments are shown.

The melanosome is a special subcellular organelle in melanocytes that synthesizes melanin [29]. The variance of skin pigment is primarily determined by the quantity, composition, maturation, and distribution of melanosomes. Its maturation process can be classified into four stages: Stage I and stage II—mainly the formation of the melanosome matrix without melanin; Stage III—initial melanin deposition (30–50% matrix filling); Stage IV—fully melanized organelles (>95% density) with characteristic electron-opaque appearance [29]. Ultrastructural assessment revealed potential effects of SAL on melanosome development: (1) a higher proportion of early-stage (Stage I/II) vesicles; (2) an observable decrease in melanosome count (Figure 3A). These morphological changes suggest that SAL could affect melanosome biogenesis, contributing into the decreased melanin synthesis.

The immunofluorescence staining also showed that SAL hindered the formation of melanosomes in melanocytes (HMB45, a melanosome marker). The melanosomes in SP/Cort group were structurally dispersed, whereas most melanosomes in the SAL group were clustered around the nucleus (Figure 3B,C). These results reveal that SAL specifically blocks centrifugal melanosome movement and terminal transfer to keratinocyte-targeting dendrites. 

### 2.4. SAL Inhibits SP/Cort-Induced Hyperpigmentation in Zebrafish and C57BL/6 Mice

**Figure 4:**
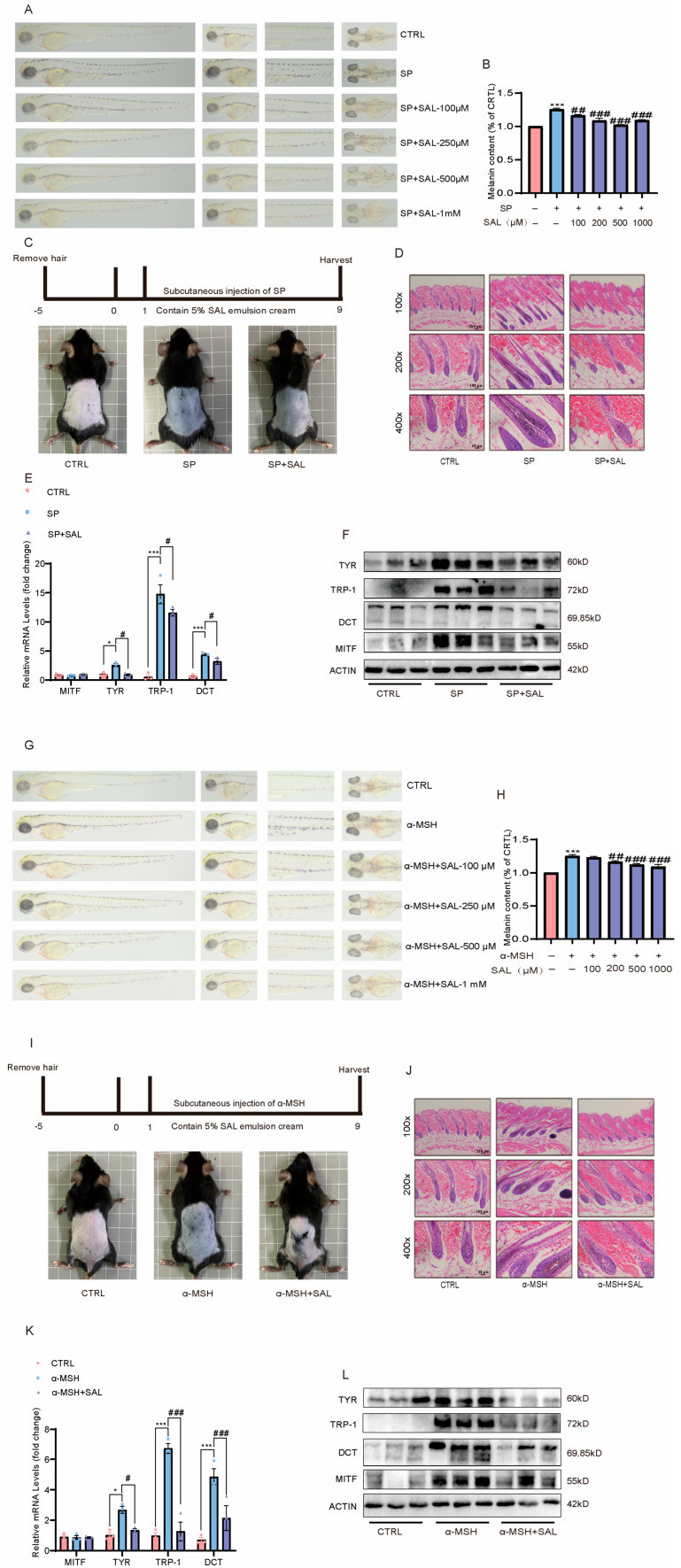
SAL inhibits SP/Cort- and α-MSH-induced hyperpigmentation in zebrafish and C57BL/6 mice. (**A**–**F**) SP/Cort-induced model: (**A**) Representative images of melanin pigmentation in zebrafish (treated with PTU). Scale bar = 1 mm. (**B**) Quantitative analysis of melanin particle area in zebrafish (n = 20 fish/group). (**C**) Dorsal skin color images of C57BL/6 mice. (**D**) Representative H&E staining of mouse hair follicles. scale bar: 200 μm for 100×, 100 μm for 200×, and 50 μm for 400× magnification.(**E**) Cutaneous mRNA expression levels of *Mitf, Tyr*, *Trp-1*, and *Dct* in mice (*n* = 5/group). (**F**) Western blot analysis of MITF, TYR, TRP-1, and DCT protein expression in mouse dorsal skin. (**G**–**L**) α-MSH-induced model:(**G**) Representative zebrafish pigmentation images. (**H**) Quantification of melanin area in zebrafish (*n* = 20/group). (**I**) Dorsal skin color images of mice. (**J**) H&E staining of hair follicles. (**K**) Cutaneous mRNA expression of melanogenic genes in mice (*n* = 5/group). (**L**) Western blot analysis of melanogenic proteins. Data are presented as mean ± SEM. * *p* < 0.05, *** *p* < 0.001 vs. CTRL are considered significant. # *p* < 0.05, ## *p* < 0.01, and ### *p* < 0.001 vs. (SP or α-MSH) are considered to be significant. Representative image from three independent experiments are shown.

The inhibitory effect of SAL on SP/Cort and α-MSH-induced pigmentation in zebrafish was investigated. Quantitative analyses revealed that both SP/Cort and α-MSH induced pronounced pigmentation across zebrafish body surfaces, whereas SAL treatment significantly attenuated these melanogenic effects (Figure 4A,B,G,H). Additionally, SP/Cort and α-MSH were utilized to induce skin dorsal pigmentation on C57BL/6 mice. After 9 days, marked dorsal hyperpigmentation was observed in the model group, while SAL administration resulted in a significant reduction (Figure 4C,I). Histological analysis via HE staining revealed melanin accumulation in the hair shaft and dermal papilla of the model group, which was robustly suppressed by SAL treatment (Figure 4D,J). RT-qPCR and Western blot analyses furtherly confirmed that the SP/Cort and α-MSH-induced melanogenic program, characterized by elevated TYR, TRP-1, and DCT expression, was substantially repressed by SAL treatment (Figure 4E,F,K,L).

These results demonstrate the efficacy of SAL in attenuating stress-induced pigmentation in vivo. It should be noted that the zebrafish model utilized PTU pre-treatment to establish a low-pigmentation baseline. While this is a standard practice to enhance the dynamic range for observing pigmentation induction and inhibition, we acknowledge that PTU, as a tyrosinase inhibitor, may alter the cellular context and potentially influence the absolute sensitivity of the system to SAL. Therefore, the quantitative potency of SAL observed in this model should be interpreted within this specific experimental framework. Nevertheless, the consistent inhibitory trend across zebrafish, mouse, and ex vivo human skin models strongly supports the core conclusion that SAL possesses anti-melanogenic activity.

### 2.5. SAL Can Bind a Novel Melanogenic Target SEC23A Through Drug Affinity Responsive Target Stability (DARTS) Screening

**Figure 5:**
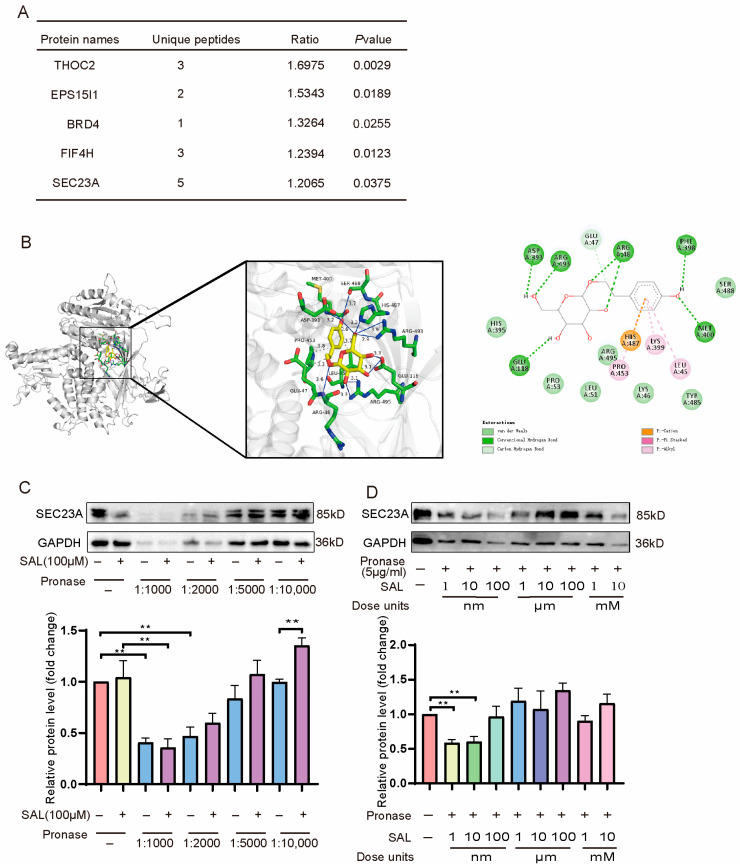
SAL can bind a novel melanogenic target SEC23A through “DARTS” screening. (**A**) Top candidate proteins enriched in SAL-treated DARTS samples; (**B**) SAL exhibited good binding activity to SEC23A as determined by molecular docking; (**C**,**D**) SAL treatment decreases the protease susceptibility of SEC23A in cell lysates, as determined by the DARTS assay, indicating that SAL stabilizes SEC23A by direct binding. For graphical representation, data are presented as mean ± standard error of the mean (*n* = 3 per group). ** *p* < 0.01 compared with the control group. Representative images from three independent experiments are shown.

To investigate potential targets for SAL, we first identified SAL-binding proteins using a label-free strategy known as “DARTS” (Drug Affinity Responsive Target Stability) [30]. We comprehensively screened and analyzed SAL-binding proteins in B16F10 cells using liquid chromatography-mass spectrometry (LC-MS). THOC2, EPS15I1, BRD4, FIF4H, and SEC23A were identified as candidate targets with high variability among the SAL-treated samples (Figure 5A). Then, molecular docking results showed that the SAL docking score of THOC2, EPS15I1, BRD4, FIF4H, and SEC23A was −7.1, −5.7, −7.3, −6.0, and −8.3 (Figure 5B, Supplementary Figure S3A–D). We further validated the top two proteins (BRD4 and SEC23A) in the molecular docking score. The DARTS assay is also a ligand-target binding confirmation technology based on the reduced protease susceptibility of the target protein after binding to the ligand. In Figure 5C, our result showed that SAL stabilized SEC23A in cell lysate after treatment with protease (1–10 μg/mL) (Figure 5C). Subsequent dose–response experiments (1 nM^−1^ mM SAL with 5 μg/mL protease) revealed maximal stabilization efficacy at 100 μM (Figure 5D). Notably, this effect was specific to SEC23A, as BRD4 protein levels remained unaffected by SAL co-treatment with protease (Supplementary Figure S3E). These collective findings strongly support direct SAL-SEC23A binding.

### 2.6. SAL Can Directly Interact with Up-Regulated Affluent SEC23A to Inhibit Hyperpigmentation in SP/Cort-Induced Model

**Figure 6:**
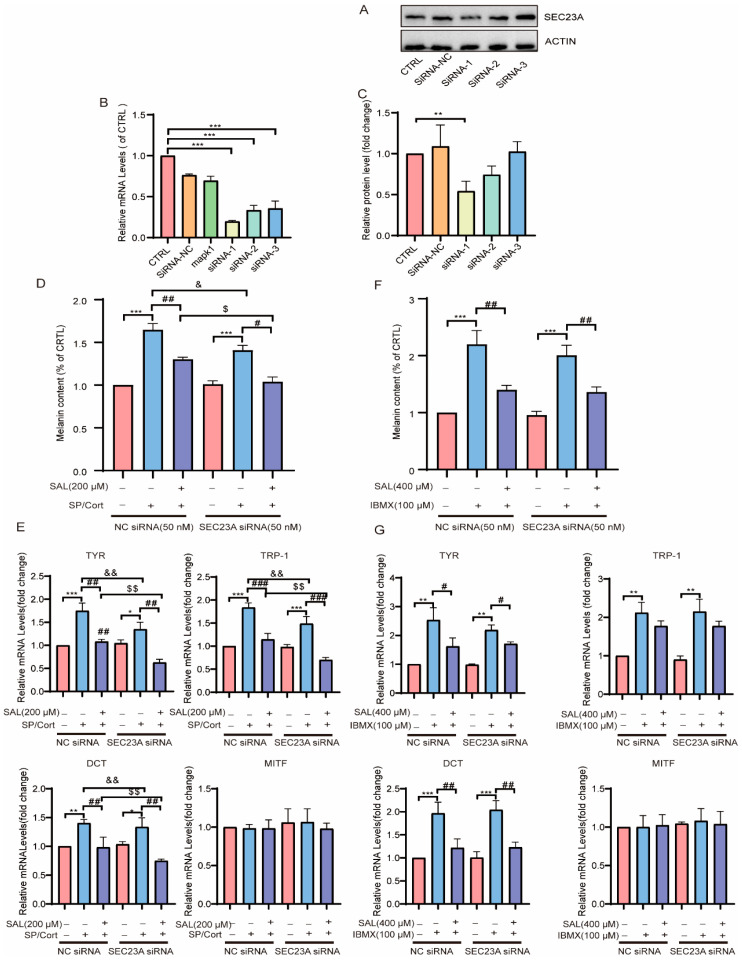
SAL can directly interact with up-regulated affluent SEC23A to inhibit hyperpigmentation in SP/Cort-induced model. (**A**) The mRNA expression of *Sec23a* was measured in siRNA-transfected cells; (**B**,**C**) the protein expression of SEC23A was measured in siRNA-transfected cells; (**D**) the effect of SAL (50, 100, and 200 µM for 72 h) on melanin contents in siRNA-transfected cells in the SP/Cort model. (**E**) the effect of SAL on the mRNA expression levels of *Mitf*, *Tyr*, *Tyr-1*, and *Dct* in siRNA-transfected cells in the SP/Cort model; (**F**) melanin contents in siRNA-transfected cells after SAL treatment different concentrations of 50, 100, and 200 µM for 72 h in the IBMX model; (**G**) the effect of SAL on the mRNA expression levels of *Mitf*, *Tyr*, *Tyr-1*, and *Dct* in siRNA-transfected cells in the IBMX model. For graphical representation, data are presented as mean ± standard error of the mean (*n* = 3 per group. * *p* < 0.05, ** *p* < 0.01, and *** *p* < 0.001 vs. CTRL are considered significant. # *p* < 0.05, ## *p* < 0.01, and ### *p* < 0.001 vs. (SP/Cort or IBMX) are considered to be significant. ^&^ *p* < 0.05, ^&&^ *p* < 0.01, vs. (NC group SP/Cort or IBMX) are considered to be significant. ^$^ *p* < 0.05, ^$$^ *p* < 0.01, vs. (NC group SAL) are considered to be significant. Representative images from three independent experiments are shown.

Our above investigation has established SEC23A as a critical mediator of SAL’s anti-pigmentation effects. Following SEC23A siRNA transfection in B16F10 melanocytes, we quantitatively assessed silencing efficacy through WB and qPCR assays (Figure 6A–C). The results showed that SEC23A knockdown was achieved using siRNA-1 (50 nM) (Figure 6A–C). The melanin content was decreased in SEC23A knockdown cells (Figure 6D). When added with SAL, its depigmenting effect was further potentiated after SEC23A knockdown in the SP/Cort model (Figure 6D). Consistently, genetic silencing of SEC23A substantially suppressed mRNA expression of core melanogenic enzymes when SAL was administered in the SP/Cort-induced model (Figure 6E). By contrast, in the IBMX-induced model, SEC23A knockdown presented no alterations in melanin content or melanogenic enzyme gene levels after SAL treatment (Figure 6F,G).

**Figure 7:**
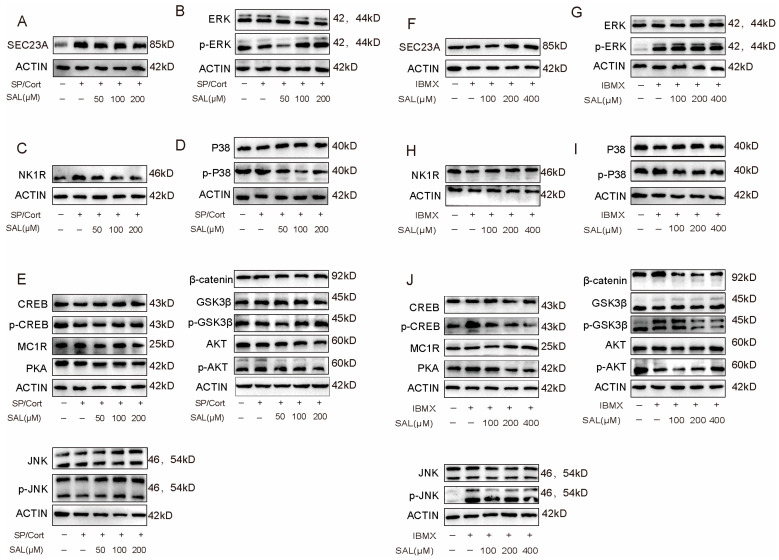
SAL exerts anti-pigmentation effects via SEC23A-related signaling specifically in the SP/Cort model, distinct from canonical cAMP pathways. (**A**–**E**) SP/Cort-induced model: (**A**) SEC23A protein expression after SAL treatment (72 h). (**B**) Phosphorylation levels of ERK. (**C**) NK1R protein expression. (**D**) Phosphorylation levels of p38. (**E**) Protein expression of MC1R, PKA, p-CREB/CREB, β-catenin, p-AKT/AKT, and p-GSK3β/GSK3β. (**F**–**J**) IBMX-induced model: (**F**) SEC23A protein expression. (**G**) ERK phosphorylation. (**H**) NK1R protein expression. (**I**) p38 phosphorylation. (**J**) Expression of MC1R, PKA, p-CREB/CREB, β-catenin, p-AKT/AKT, and p-GSK3β/GSK3β. For graphical representation, data are presented as mean ± standard error of the mean (*n* = 3 per group). Representative image from three independent experiments is shown.

We further explored the mechanistic distinction between neuroendocrine (SP/Cort) and cAMP-driven (IBMX) pigmentation to determine whether SEC23A-related melanogenic signaling, specifically responsive to neuroendocrine stress (SP/Cort), is independent of canonical cAMP pathways (IBMX). Firstly, we observed that SP/Cort challenge significantly upregulated SEC23A expression, and SAL treatment normalized this elevation (Supplementary Figure S3A). However, in the IBMX-induced model, SEC23A levels remained unchanged (Supplementary Figure S3D). The detailed protein expression of SEC23A in both models is further illustrated in Figure 7A,F.

### 2.7. SAL Has an Anti-Pigmenting Effect Through SEC23A-Related Signaling Axis Responsive to SP/Cort, Different from Canonical cAMP Pathways (IBMX)

To gain insights into potential mechanisms, we investigated the effect of SAL on melanogenic signaling pathways. In the SP/Cort-induced model, SEC23A expression was abnormally up-regulated and then down-regulated by SAL treatment (Figure 7A, Supplementary Figure S4A). Besides, we used a variety of chemical-physical methods to validate that SAL could directly bind to SEC23A (Figure 5B–D). It’s reported that a novel mechanism involving “SEC23A-PF4-ERK/MAPK axis” inhibits melanoma metastasis [31]. Therefore, we examined whether the downstream MAPK pathway was activated. We found that SP/Cort significantly downregulated ERK phosphorylation, and SAL was able to significantly upregulate its phosphorylation (Figure 7B, Supplementary Figure S4A). Meanwhile, the NK1R protein, which has the highest affinity for SP, was significantly elevated in the model group, and SAL effectively reversed this trend (Figure 7C, Supplementary Figure S4B). Previous studies have shown that SP activates NK1R and regulates the p38 MAPK pathway [32], which regulates MITF expression, thus leading to the pigmentation process. As predicted, the phosphorylation level of p38 was upregulated in SP/Cort-induced hyperpigmentation, and this phenomenon could be reversed after SAL intervention (Figure 7D, Supplementary Figure S4B). However, protein levels of MC1R (the receptor for α-MSH) and PKA remained unaltered (Figure 7E, Supplementary Figure S4C). It has been shown that the phosphorylated GSK3β protein in the PI3K/AKT pathway interacts with and phosphorylates MITF [33], thereby regulating the expression of related downstream proteins. We examined the PI3K/AKT signaling pathway and found no effect in the SP/Cort model (Figure 7E, Supplementary Figure S4C). The classical Wnt pathway induces the accumulation of β-catenin in the melanocyte cytoplasm and its entry into the nucleus, where it binds to LEF1 and acts at the MITF promoter site to enhance its transcriptional expression, thereby affecting melanin synthesis [34]. We examined the protein expression levels of β-catenin in the stressful factor SP/Cort model and showed no change (Figure 7E, Supplementary Figure S4C). Our integrated analyses reveal that SAL regulates a dual-axis pathway network through SEC23A engagement to counteract stress-induced hyperpigmentation: (1) “ERK-MITF axis” and (2) “NK1R-P38 -MITF axis”.

In contrast, in the IBMX (canonical cAMP pathways)-induced model, neither IBMX nor SAL had any effect on SEC23A expression (Figure 7E, Supplementary Figure S5A). Despite p-ERK protein expression being upregulated in the IBMX-induced model, SAL can’t reverse this (Figure 7G, Supplementary Figure S5A). Meanwhile, we found that protein levels of p-p38 were elevated in the model and decreased significantly with SAL intervention (Figure 7I, Supplementary Figure S5A); however, protein levels of NK1R were examined and did not change (Figure 7H, Supplementary Figure S5B). Nevertheless, the level of MC1R protein was found to be significantly increased in model group, and SAL was able to reverse this result. SAL downregulated the expression of PKA, inhibited the phosphorylation of CREB (Figure 7J, Supplementary Figure S4C), and downregulated the expression of p-AKT, including GSK3β/β-catenin (Figure 7J, Supplementary Figure S4C). These data demonstrate that SAL inhibits IBMX-induced melanin production by regulating multiple pigmentary pathways, including wnt/β-catenin, PKA/CREB, and PI3K/AKT pathways. These comparative analyses demonstrate fundamental differences in SAL’s anti-melanogenic action between the SP/Cort-induced neuroendocrine stress model and the IBMX-induced canonical cAMP model.

## 3. Discussion

The significance of the present study are threefold: First, it is the first to apply SAL to a psychological stress-induced pigmentation model, demonstrating its inhibitory effect on neuroendocrinologically-mediated aberrant melanogenesis and thus expanding the potential application scenarios of SAL. Second, it is the first to identify SEC23A as a core regulatory target in stress-induced pigmentation, demonstrating that SAL inhibits the dual signaling axes of “SEC23A-p-ERK1/2” and “NK1R-p-P38” via selective binding to SEC23A, thereby addressing the knowledge gap regarding the “downstream targets and intervention mechanisms of stress-mediated pigmentation. Third, it clarifies the mechanistic differences in SAL between the stress model (SEC23A-dependent) and the classic model (PKA/CREB, PI3K/AKT pathway-dependent), providing an experimental basis for subsequently “developing precise intervention strategies for pigmentation disorders with different etiologies.” In summary, this study not only offers a new perspective on the etiology of psychological stress-related pigmentation diseases but also lays the foundation for SAL as a candidate therapeutic molecule for such conditions.

Melanogenesis is critical for UV protection; however, its dysregulation results in pigmentation disorders, which account for approximately 8% of dermatological consultations [35]. Over the past decade, the field of dermatology has increasingly recognized that psycho-neuroendocrine axes, via neuromediators and the HPA axis, can be key drivers of pigmentation disorders [8,36]. Through multi-dimensional experiments in vitro, in vivo, and in skin tissue, we observed that SAL dose-dependently reduced SP/Cort-induced melanin deposition in B16F10 cells at 200 μM, a concentration significantly lower than the 400 μM required in the classic IBMX-induced cAMP model (Figure 1). Ex vivo experiments confirmed that SAL reversed SP/Cort-induced pigment deposition at the dermal–epidermal junction in human foreskin tissue (Figure 2A). In vivo validation further demonstrated that SAL alleviated SP/Cort- and α-MSH-induced pigmentation in zebrafish and inhibited hair follicle melanin accumulation in C57BL/6J mice, while downregulating TYR, TRP-1, and DCT expression (Figure 4). Additionally, TEM and HMB45 immunofluorescence revealed that SAL not only reduced melanosome numbers but also corrected their abnormal retention at stage III (Figure 3). In contrast to the stress model, UVB and α-MSH collaboratively construct a physiological melanosome regulatory network through complementary, receptor-mediated signaling: UVB activates the MC1R/cAMP-PKA pathway in melanocytes, promoting melanosome biogenesis, while α-MSH upregulates exocytosis-related molecules, triggering melanosome transfer to keratinocytes [37]. This synergy coordinates physiological pigmentation associated with skin photoprotection. In our stress model, melanosomes exhibited pathological characteristics—aberrant proliferation, hypermaturation, and impaired transport—accompanied by SEC23A upregulation. SAL corrected these phenotypes by selectively binding SEC23A, underscoring its target specificity and pathological relevance.

Mechanistically, SEC23A, a core subunit of the COPII complex, mediates ER-to-Golgi transport of secretory proteins, a process crucial for melanosome maturation [38,39,40]. Through DARTS assays and molecular docking screening, SEC23A was identified as the core target for SAL’s intervention, with the highest docking score (−8.3) and enhanced protease resistance upon SAL binding (Figure 5). SP/Cort stress significantly upregulated SEC23A expression, which SAL normalized (Figure 7A). SAL concurrently regulated two key signaling pathways: (1) activation of the SEC23A-p-ERK-MITF axis—SAL reversed SP/Cort-induced downregulation of p-ERK, promoting MITF degradation (without affecting its mRNA level) and thereby inhibiting TYR, TRP-1, and DCT expression (Figure 1D,H and Figure 7B); and (2) inhibition of the NK1R-p38-MITF axis—SAL reversed SP/Cort-induced upregulation of NK1R and p38 phosphorylation, indirectly regulating melanogenic protein synthesis and transport (Figure 7C,D). This dual regulation simultaneously targets melanin synthesis and melanosome maturation, forming a synergistic inhibitory effect that attenuates the pigmentation phenotype.

Notably, the anti-pigmentation effect of SAL exhibits significant etiological specificity, with distinct mechanisms in the SP/Cort stress model compared to the classic IBMX-induced cAMP model. In the SP/Cort model, SEC23A is essential for SAL’s action; siRNA-mediated SEC23A knockdown significantly reduced melanin deposition, and SAL further enhanced this inhibitory effect (Figure 6D,E). Conversely, SAL had no impact on canonical pathways like Wnt/β-catenin, PI3K/Akt, or PKA/CREB in this model (Figure 7E). In the IBMX model, SEC23A expression remained unchanged, and SAL acted by directly regulating downstream cAMP signaling—downregulating PKA expression, inhibiting CREB phosphorylation, and modulating GSK3β/β-catenin and PI3K/Akt pathways (Figure 7F–J). This contrasts with the UVB model, where SAL targets P4HB and regulates the IRF1/USF1 transcriptional complex [22], further underscoring SAL’s context-dependent, multi-target properties. This target dependency is also reflected in potency: SAL achieved significant effects at 200 μM in the stress model but required 400 μM in the IBMX model. KEGG analysis supported this disparity, showing SAL primarily enriched “Melanogenesis pathway” genes in the SP/Cort model, while enriching PI3K/AKT, MAPK, and cAMP pathways in the IBMX model (Figure 2C,E).

This study advances current knowledge in two key aspects, thereby refining the theoretical framework governing the stress-pigmentation axis. First, it expands the target profile and application potential of SAL. Previous research only confirmed that SAL targets P4HB in UVB-induced models [22]; our study is the first to discover that SAL acts through a novel target, SEC23A, in psychological stress models. The distinct nature of SEC23A from P4HB indicates that SAL possesses multi-target and context-adaptable properties, challenging the conventional one-target-fits-one-disease paradigm. Second, this work establishes a new regulatory function for SEC23A as a critical node in stress-mediated excessive melanogenesis. Previously considered primarily a structural protein for melanosome transport, we demonstrate that SEC23A serves as a downstream effector for neuroendocrine stress signals, integrating SP and Cort signaling to modulate both ERK and p38 MAPK pathways. Building on our team’s prior finding that SP exacerbates Cort-induced pigmentation [23], this study identifies SEC23A as the crucial downstream target for this signal convergence, completing the regulatory pathway from upstream triggers to downstream effects.

An intriguing finding is the context-dependent dual regulation of MITF and ERK signaling by SAL. In the SP/Cort model, SAL downregulated MITF protein expression while unexpectedly upregulating p-ERK/ERK levels (Supplementary Figure S4). This apparent paradox can be explained by the intensity- and context-dependent outcomes of ERK signaling [41,42]. Transient versus sustained ERK activation can elicit opposing cellular responses; moderate ERK activation may trigger adaptive negative feedback loops or promote MITF phosphorylation-dependent ubiquitination and degradation [43,44], ultimately suppressing melanogenesis. This “reprogrammed” ERK action, specific to the stress context, contrasts with SAL’s effects in the IBMX model and further exemplifies its etiological specificity. Such flexibility may enable SAL to precisely target stress-induced hyperpigmentation without broadly disrupting normal melanogenic programs.

Several limitations of the present work should be acknowledged. First, the use of B16F10 mouse melanoma cells, while valuable for melanogenesis research, may not fully recapitulate primary human melanocyte biology; future studies should utilize primary human melanocytes and 3D skin reconstructs. Second, although DARTS and siRNA experiments implicate SEC23A as a direct SAL target, rescue experiments (e.g., SEC23A overexpression) are needed to definitively establish causality. However, a very recent independent study [45] provides compelling functional evidence for the role of Sec23a in melanogenesis, demonstrating that the BBF2H7-Sec23a axis is essential for COPII-mediated anterograde transport of tyrosinase from the endoplasmic reticulum to melanosomes. This external validation supports the biological plausibility of our proposed mechanism and reinforces SEC23A as a critical regulator of melanin synthesis. Nevertheless, future studies incorporating overexpression and genetic rescue strategies would further solidify the direct functional link between SEC23A and the anti-pigmentation activity of SAL. Third, the precise mechanisms underlying MITF downregulation (e.g., ubiquitination) remain to be elucidated, as we did not perform half-life or ubiquitination assays. Fourth, the human foreskin organ culture assessed only short-term (1-week) effects, lacking long-term safety data. Fifth, the absence of clinical samples from patients with stress-exacerbated pigmentary disorders (e.g., melasma with anxiety) limits translational validation. Future research incorporating human-relevant models, genetic rescue strategies, and clinical cohorts is warranted to address these gaps.

In conclusion, this study identifies SEC23A as a novel core target in psychological stress-induced hyperpigmentation and demonstrates that SAL, through selective SEC23A binding, modulates the dual signaling axes of SEC23A-p-ERK-MITF and NK1R-p38-MITF. This action significantly inhibits stress-induced hypermelanogenesis and pathological melanosome maturation, with strict specificity to the neuroendocrine stress model—fundamentally differing from the PKA/CREB/PI3K pathway-dependent mechanism observed in canonical cAMP-driven pigmentation. These findings refine the molecular framework of stress-regulated melanogenesis, establish SEC23A as a critical node for therapeutic intervention, and provide a novel experimental foundation for the precise treatment of pigmentary diseases.

## 4. Materials and Methods

### 4.1. Cell Culture

B16F10 mouse melanoma cells were cultured in Dulbecco’s modified Eagle’s medium (DMEM; meilunbio, Dalian, Liaoning, China) containing 10% fetal bovine serum (FBS, meilunbio) and antibiotic-antimycotic solution (meilunbio) at 37 °C in a 5% CO_2_ atmosphere. The B16F10 cell line used in this study was obtained from the Cell Bank of the Chinese Academy of Sciences (Shanghai, China).

### 4.2. Melanin Content Measurement

Melanin content was measured via a previously described method [46]. Cells were treated with 3-Isobutyl-1-methylxanthine (isobutylmethylxanthine, IBMX, 100 μM) plus SAL (100, 200, 400 μM) (MCE, MedChemExpress, Monmouth Junction, NJ, USA) or substance P (SP; 10 nM)/cortisol (Cort,10 μM) plus SAL (50, 100, 200 μM) for 72 h. Cell pellets were dissolved in 100 μL 1 N NaOH/10% DMSO, incubated at 80 °C for 2 h, and absorbance was measured at 405 nm using a microplate reader (BioTek Instruments, Winooski, VT, USA).

### 4.3. Cellular Tyrosinase Activity Assay

Tyrosinase activity was determined by measuring the L-DOPA oxidation rate [46]. Cells were lysed with cell lysis buffer (containing 1% PMSF), and protein content was quantified by a BCA protein assay kit (Beyotime Institute of Biotechnology, Shanghai, China). A 100 μL mixture of PBS (pH 6.8) containing 20 μg protein and 100 μL 0.01% L-DOPA was incubated at 37 °C for 1 h in the dark, and absorbance was measured at 475 nm (BioTek Instruments, USA).

Hydroquinone (HQ; purity ≥ 99%, Sigma-Aldrich, St. Louis, MO, USA) was used as a positive control in melanin content and tyrosinase activity assays. HQ was dissolved in DMSO and diluted in culture medium to a final concentration of 10 μM, with a final DMSO concentration below 0.1%.

### 4.4. Mice Experiment

All animal experiments complied with Laboratory Animal Care guidelines and were approved by the Faculty Animal Committee, Shanghai University of Traditional Chinese Medicine (Permit No.: PZSHUTCM2303050004; Approval Date: 5 March 2023). SAL (0.5%, *w*/*w*) was dissolved in an oil-in-water emulsion cream (61.3% water, 8.0% stearic acid, 8.0% white Vaseline, 7.0% glycerol, 6.0% octadecanol, 5.0% propylene glycol, 2.0% azone, 1.6% trolamine, 1.0% sodium dodecylsulfate, 0.1% ethylparaben). A commercial compound dexamethasone acetate cream (Baiyunshan, Guangzhou, China; containing 0.75 mg dexamethasone acetate per gram, i.e., 0.075% *w*/*w*) was used as a reference topical corticosteroid. Male C57BL/6 mice (6 weeks old; Charles River, Shanghai, China) were housed under controlled conditions (12-h light/dark cycle, 23 ± 1 °C, 50% humidity) with ad libitum food/water. After 1 week of acclimation, mice were depilated and randomized into 5 groups: control (emulsion cream only), model 1 (daily subcutaneous injection of 0.15 mM α-MSH) [47], model 2 (daily subcutaneous injection of 0.08088 mg/kg/d SP plus topical application of the dexamethasone acetate cream), SAL group 1 (0.15 mM α-MSH + 0.5% SAL cream), and SAL group 2 (0.08088 mg/kg/d SP plus topical application of 0.5% SAL cream and the dexamethasone acetate cream). Treatments were applied to the dorsal skin for 8 days, with images captured on day 9.

### 4.5. Skin Tissue Staining

Human foreskin specimens were obtained from healthy pediatric donors (aged 6–14 years) undergoing elective circumcision, with ethical approval granted by the Urology Department of Shanghai Children’s Hospital. The cultured normal human skin tissues were divided into three groups: a control group (no treatment), a model group treated with SP (100 nM) + Cort (100 μM), and a treatment group receiving SAL (400 μM) + SP (100 nM) + Cort (100 μM). All groups were incubated for one week at 37 °C under 5% CO_2_. Subsequently, the tissues were fixed in 4% paraformaldehyde, dehydrated, embedded in paraffin, and sectioned into 5 μm slices. Sections were stained using Masson–Fontana dye (Sbjbio, BP-DL371, Nanjing, Jiangsu, China) according to the manufacturer’s protocol. Imaging was performed using a microscope (OLYMPUS, BX43F, Tokyo, Japan).

Mouse dorsal skin samples were similarly fixed in 4% paraformaldehyde, embedded in paraffin, sectioned at 5 μm, and stained with hematoxylin and eosin (Servicebio, Wuhan, Hubei China) following the manufacturer’s instructions. Images were acquired using the same microscope model (OLYMPUS BX43F, Center Valley, PA, USA).

### 4.6. Zebrafish Experiment

All procedures adhered to ethical guidelines to minimize animal suffering and were approved by the Ethics Committee of Shanghai Tong Ren Hospital (Approval Code: Tong Ren Ethics Approval A2025-063-01; Approval Date: 18 November 2025). Zebrafish embryos were obtained from the Hongqiao International Institute of Medicine, Shanghai Jiao Tong University School of Medicine (Shanghai, China), with reproduction methods as described [48]. To synchronize embryonic development and facilitate the visualization of induced pigmentation in subsequent steps, embryos were treated with 50 μg/mL N-phenylthiourea (PTU), a tyrosinase inhibitor, from 6 to 72 h post-fertilization (hpf) to suppress baseline melanogenesis. Following extensive washing to remove PTU, 72 hpf larvae were then randomly allocated into experimental groups. Model groups were treated with SP (10 nM)/(Cort,10 μM) or α-MSH (100 μM) for 48 h, alongside SAL (100, 250, 500, 1000 μM) for the drug group. Images were captured using an Olympus stereoscope and quantified with ImageJ 1.8.0.

### 4.7. Immunofluorescence Staining and Transmission Electron Microscopy

B16F10 cells were seeded on poly-Lys-coated glass slides, fixed with p-formaldehyde, and permeabilized with PBS containing 0.5% Triton X-100 (Biosesang, PR4007, Sungnam, Republic of Korea) for 10 min. Cells on glass slides were incubated with PBS containing 5% bovine serum albumin (BSA; Sigma-Aldrich, A9647) and reacted with an antibody against HMB45. After washing, cells on glass slides were stained with Alexa Fluor 562-conjugated secondary antibody (Thermo Fisher Scientific, A-11034, Delaware, DE, USA) and also with Vectashield antifade medium containing 4′,6-diamidino-2-phenylindole (DAPI; Vector Lab, California, CA, USA), and examined using a confocal microscope (Carl Zeiss LSM 980).

Cells were treated with IBMX (100 μM) and SAL (400 μM) or SP (10 nM)/Cort (10 μM) and SAL (200 μM) for 72 h; the cells were collected and fixed in 2.5% (V/V) glutaraldehyde. The fixed cells were washed thrice with buffer for 10–20 min, after which they were fixed again in 1% osmium acid, dehydrated with a gradient of ethanol, embedded in paraffin, and stained. Images were captured using a transmission electron microscope (Tecnai G2 Spirit BioTWIN; FEI, Hillsboro, OR, USA).

### 4.8. Western Blot Analysis

Protein extracts were prepared from mice dorsal skin tissues, B16F10 cells, resolved on sodium dodecyl sulfate (SDS)-acrylamide gels by electrophoresis, and transferred to the membranes of PVDF using a semidry blotting apparatus. After blocking with 5% non-fat milk (BD Biosciences, Monmouth Junction, NJ, USA) in Tris-buffered saline (TBS) with Tween 20, blots were incubated with primary antibody overnight at 4 °C. After washing with TBS containing Tween 20, blots were incubated with horseradish peroxidase (HRP)-labeled secondary antibody for 1–3 h and visualized the immune complex by a chemiluminescence kit (GE Healthcare, Chicago, IL, USA, RPN2232). Primary antibodies were anti-Actin (Abcam, ab7817, Cambridge, UK); anti-MITF-M (Abcam, ab20663); anti-TYR (Abcam, ab180753); anti-glyceraldehyde 3-phosphate dehydrogenase (GAPDH; Abcam, ab8245); anti-p-CREB (Cell Signaling Technology, 9198); anti-CREB (Cell Signaling Technology, 9197, Massachusetts, MA, USA); anti-β-catenin (Cell Signaling Technology, 9567); anti-GSK (Cell Signaling Technology, 9562); anti-p-GSK (Cell Signaling Technology, 79774); anti-AKT (Cell Signaling Technology, 9272); anti-p-AKT (Cell Signaling Technology, 4060), anti-p-p38 (Cell Signaling Technology, 9211); anti-p38MAPK (Cell Signaling Technology, 9212); anti-p-ERK (Cell Signaling Technology, 9101); anti-ERK (Cell Signaling Technology, 9102); anti-p-JNK (Cell Signaling Technology, 9251); anti-JNK (Cell Signaling Technology, 9252); anti-MC1R (Cell Signaling Technology, 9595); anti-PKA (Abcam, ab32514); anti-NK1R (Abcam, ab75516); anti-tyrosinase (TYR)-related protein-1 (TRP-1; Abcam, ab235447); and anti-dopachrome tautomerase (DCT; Abcam, ab221144); anti-SEC23A (abclonal, A8613, Wuhan, Hubei, China). Secondary antibodies were HRP-labeled anti-rabbit IgG (Thermo Fisher Scientific, 31460) and HRP-labeled anti-mouse IgG (Thermo Fisher Scientific, 31430).

### 4.9. Quantitative Reverse Transcription-Polymerase Chain Reaction (PCR)

Total RNAs were extracted from B16F10 cells using a TRIZOL kit. Complementary DNA (cDNA) was prepared from 1 μg of total RNA using a reverse transcription kit and subjected to PCR analysis to determine the levels of each transcript, such as *Tyr*, *Trp-1*, *Dct*, *Mitf*, and *Sec23a*. Nucleotide sequences of PCR primers were described in Table S1. RT-PCR conditions were as follows: reverse transcription at 95 °C for 5 min followed by 30 cycles of PCR at 94 °C for 30 s (denaturation), 50–60 °C for 1 min (annealing), and 72 °C for 1 min (extension).

### 4.10. Molecular Docking

AutoDock Vina 1.1.2 was used for molecular docking. The 3D structure of SAL was downloaded from PubChem. Protein structures (THOC2, FPS15I1, BRD4, FIF4H, SEC23A) were obtained from the RCSB database. PyMOL 4.3.0 (https://pymol.org/, accessed on 25 January 2026) removed organic matter, and Autodock Tools (http://mgltools.scripps.edu/downloads, accessed on 19th January 2025) prepared proteins (hydrogenation, charge checking, AD4 atom typing) and ligands (root assignment, flexible bond selection). Structures were converted to PDBQT format. Docking scores were calculated, and interactions were visualized using PyMOL and Discovery Studio Client v21.1.0.20298.

### 4.11. Drug Affinity Responsive Target Stability (DARTS) Assay

B16F10 cells were lysed with NP40 buffer and centrifuged (12,000 rpm, 15 min). Lysates were diluted with TNC buffer (50 mM Tris-HCl [pH 8.0], 50 mM NaCl, 10 mM CaCl_2_), divided into two aliquots, and treated with DMSO or SAL (100 μM) for 1 h at room temperature. Aliquots were digested with pronase (10, 5, 2, 1 μg/mL) at 37 °C for 20 min. Proteins were denatured with 5× loading buffer, and SEC23A/BRD4 expression was detected by Western blot.

### 4.12. Small Interfering (si) RNA Assay

The siRNAs against *Sec23a* were supplied from Genomeditech (Shanghai, China), and their nucleotide sequences were 5′-GUGCAGUUUUGAAUCCUUUTTAAAGGAUUCAAAACUGCACTT-3′ (*Sec23a*-si-1), 5′-GCUUUGGUUGGACUUAUUATTUAAUAAGUCCAACCAAAGCTT-3′ (*Sec23a*-si-2), and 5′-CAUUCGACUGUGUAAAAATTUUUUUGACACAGUCGAAUGTT-3′ (*Sec23a*-si-3). B16F10 cells were transfected with each siRNA using a lipofectamine kit, incubated for 5 h, and stimulated with IBMX (100 μM) or SP (10 nM)/Cort (10 μM) for 72 h. Total RNAs were subjected to RT-PCR analysis of *Sec23a* as described above.

### 4.13. RNA Sequencing (RNA-seq) Transcriptome Analysis

Cultured normal human foreskin (ethnic Han/aged 18–22 years) was treated with IBMX, SP/Cort, SP/Cort + SAL (400 μM), and IBMX + SAL (800 μM) for a week at 37 °C and 5% CO_2_, with ethical approval granted by the Urology Department of Shanghai Children’s Hospital. The detailed protocols were based on our previous publication [46]. Total RNA was isolated for the construction of RNA-seq libraries. The quality of the RNA libraries was evaluated using the Agilent 2200 TapeStation (Agilent Technologies, Santa Clara, CA, USA). Library sequencing was performed on a HiSeq 4000 sequencing platform (Illumina Company, San Diego, CA, USA) by the Novogene Bioinformatics Institute (Beijing, China). Clustering was performed in a cBot cluster generation system using the TruSeq PE Cluster Kit v3-cBot-HS (Illumina).

### 4.14. Statistical Analysis

Statistical analysis was performed using GraphPad Prism 8.0 software (UK), and data were expressed as mean ± standard error of the mean. For comparisons among three or more groups, data were first assessed for normality and homogeneity of variance. Subsequently, one-way analysis of variance (ANOVA) was performed. If the ANOVA indicated a statistically significant difference (*p* < 0.05), Dunnett’s post hoc test was applied for multiple comparisons between each treatment group and the designated control group. Comparisons between two groups were performed using an unpaired, two-tailed Student’s t-test. * p < 0.05, ** p < 0.01, and *** p < 0.001 vs. control (CTRL) are considered to be significant. ^#^ *p* < 0.05, ^##^ *p* < 0.01, and ^###^ *p* < 0.001 vs. (SP/Cort or IBMX) are considered to be significant.

## 5. Conclusions

This study identifies SEC23A as a novel core target in psychological stress-induced hyperpigmentation and demonstrates that salidroside (SAL) ameliorates this condition through selective SEC23A binding. SAL concurrently modulates the SEC23A–p-ERK–MITF and NK1R–p38–MITF signaling axes, thereby inhibiting stress-induced melanin synthesis and pathological melanosome maturation. Notably, this mechanism exhibits strict specificity to the neuroendocrine stress model, fundamentally differing from the PKA/CREB/PI3K pathway-dependent mechanism observed in canonical cAMP-driven pigmentation. These findings establish SEC23A as a critical regulatory node in the stress–pigmentation axis and address the previous lack of targeted intervention strategies for stress-associated pigmentary disorders. The etiological specificity of SAL’s action positions it as a promising candidate for treating conditions such as melasma or post-inflammatory hyperpigmentation in individuals with concomitant psychological stress, providing a theoretical foundation for precision dermatotherapy.

## Data Availability

The original data presented in the study are openly available in [Zenodo] at [https://doi.org/10.5281/zenodo.18901599; https://doi.org/10.5281/zenodo.18793585, accessed on 25 January 2026)].

## Supplementary Materials

The following supporting information can be downloaded at: https://www.mdpi.com/article/10.3390/ph19030487/s1. Figure S1: Effect of SAL on cell viability in B16F10 cells. Figure S2: SAL exerts a more potent anti-hyperpigmentation effect in SP/Cort-induced than in IBMX-induced B16F10 cells. Figure S3: SAL directly binds to the novel melanogenic target SEC23A identified by DARTS screening. Figure S4: SAL inhibits hyperpigmentation via the SEC23A-p-ERK-MITF and NK1R-p38-MITF axes in the SP/Cort model. Figure S5: SAL alleviates hyperpigmentation by modulating the PKA/CREB, PI3K/AKT, and Wnt/β-catenin signaling pathways in the IBMX model. Table S1: Nucleotide sequences of PCR primers.

## Abbreviations

SP: Substance P; Cort: Cortisol; α-MSH: α-Melanocyte-Stimulating Hormone; DARTS: Drug Affinity Responsive Target Stability; RT-PCR: Reverse Transcription-Quantitative Polymerase Chain Reaction; WB: Western Blot; MITF: Microphthalmia-Associated Transcription Factor; TYR: tyrosinase; TYRP-1: TYR-related protein 1; DCT: dopachrome tautomerase; PI3K/AKT: phosphatidylinositol 3-kinase/Akt protein kinase B; GSK3β: glycogen synthase kinase 3 beta; P38: P38 MAP kinases; ERK: extracellular signal-regulated kinases; JNK/SAPK: jun amino-terminal kinases/stress-activated protein kinases; CREB: cAMP-response element binding protein; IBMX: isobutylmethylxanthine; PTU, N-phenylthiourea; KEGG, Kyoto Encyclopedia of Genes and Genomes; MAPK, mitogen-activated protein kinase.

## References

[1] Solano F. (2020). Photoprotection and Skin Pigmentation: Melanin-Related Molecules and Some Other New Agents Obtained from Natural Sources. Molecules.

[2] D’Orazio J., Jarrett S., Amaro-Ortiz A., Scott T. (2013). UV radiation and the skin. Int. J. Mol. Sci..

[3] English J.S., Dawe R.S., Ferguson J. (2003). Environmental effects and skin disease. Br. Med. Bull..

[4] Fu C., Chen J., Lu J., Yi L., Tong X., Kang L., Pei S., Ouyang Y., Jiang L., Ding Y. (2020). Roles of inflammation factors in melanogenesis (Review). Mol. Med. Rep..

[5] Ju Q., Zouboulis C.C. (2016). Endocrine-disrupting chemicals and skin manifestations. Rev. Endocr. Metab. Disord..

[6] Theoharides T.C., Stewart J.M., Taracanova A., Conti P., Zouboulis C.C. (2016). Neuroendocrinology of the skin. Rev. Endocr. Metab. Disord..

[7] Simons R.E., Zevy D.L., Jafferany M. (2020). Psychodermatology of vitiligo: Psychological impact and consequences. Dermatol. Ther..

[8] Alexopoulos A., Chrousos G.P. (2016). Stress-related skin disorders. Rev. Endocr. Metab. Disord..

[9] Skobowiat C., Slominski A.T. (2015). UVB Activates Hypothalamic-Pituitary-Adrenal Axis in C57BL/6 Mice. J. Investig. Dermatol..

[10] Slominski A., Mihm M.C. (1996). Potential mechanism of skin response to stress. Int. J. Dermatol..

[11] Slominski A., Wortsman J., Tuckey R.C., Paus R. (2007). Differential expression of HPA axis homolog in the skin. Mol. Cell Endocrinol..

[12] Zbytek B., Wortsman J., Slominski A. (2006). Characterization of a ultraviolet B-induced corticotropin-releasing hormone-proopiomelanocortin system in human melanocytes. Mol. Endocrinol..

[13] Turk T., Liu C., Straube S., Dytoc M., Hagtvedt R., Dennett L., Abba-Aji A., Fujiwara E. (2022). The global prevalence of primary psychodermatologic disorders: A systematic review. J. Eur. Acad. Dermatol. Venereol..

[14] Zhu Y., Du X., Shen S., Song X., Xiang W. (2024). Body dysmorphic disorder symptoms in patients with melasma. Int. J. Dermatol..

[15] Chen M., Cai J., Zhang X., Liao Z., Zhong M., Shang J., Yue Y. (2022). Keratinocytes take part in the regulation of substance P in melanogenesis through the HPA axis. J. Dermatol. Sci..

[16] Inoue K., Hosoi J., Ideta R., Ohta N., Ifuku O., Tsuchiya T. (2003). Stress augmented ultraviolet-irradiation-induced pigmentation. J. Investig. Dermatol..

[17] Liu N., Wang L.H., Guo L.L., Wang G.Q., Zhou X.P., Jiang Y., Shang J., Murao K., Chen J.W., Fu W.Q. (2013). Chronic restraint stress inhibits hair growth via substance P mediated by reactive oxygen species in mice. PLoS ONE.

[18] Pang S., Wu H., Wang Q., Cai M., Shi W., Shang J. (2014). Chronic stress suppresses the expression of cutaneous hypothalamic-pituitary-adrenocortical axis elements and melanogenesis. PLoS ONE.

[19] Yang S., Wang L., Zeng Y., Wang Y., Pei T., Xie Z., Xiong Q., Wei H., Li W., Li J. (2023). Salidroside alleviates cognitive impairment by inhibiting ferroptosis via activation of the Nrf2/GPX4 axis in SAMP8 mice. Phytomedicine.

[20] Zhang X., Xie L., Long J., Xie Q., Zheng Y., Liu K., Li X. (2021). Salidroside: A review of its recent advances in synthetic pathways and pharmacological properties. Chem. Biol. Interact..

[21] Chiang H.M., Chien Y.C., Wu C.H., Kuo Y.H., Wu W.C., Pan Y.Y., Su Y.H., Wen K.C. (2014). Hydroalcoholic extract of Rhodiola rosea L. (Crassulaceae) and its hydrolysate inhibit melanogenesis in B16F0 cells by regulating the CREB/MITF/tyrosinase pathway. Food Chem. Toxicol..

[22] Ding X.J., Zhang Z.Y., Jin J., Han J.X., Wang Y., Yang K., Yang Y.Y., Wang H.Q., Dai X.T., Yao C. (2020). Salidroside can target both P4HB-mediated inflammation and melanogenesis of the skin. Theranostics.

[23] Yang M., Cheng K., Gu J., Wu H.-l., Li Y.-m. (2025). Inhibitory Effects of Nardostachys Jatamansi DC. Volatile Oil on Psychological Factors SP/CORT-Induced Hyperpigmentation. Chin. J. Integr. Med..

[24] Myung C.H., Lee J.E., Jo C.S., Park J.i., Hwang J.S. (2021). Regulation of Melanophilin (Mlph) gene expression by the glucocorticoid receptor (GR). Sci. Rep..

[25] Scheau C., Draghici C., Ilie M.A., Lupu M., Solomon I., Tampa M., Georgescu S.R., Caruntu A., Constantin C., Neagu M. (2021). Neuroendocrine Factors in Melanoma Pathogenesis. Cancers.

[26] Vagnerová K., Jágr M., Mekadim C., Ergang P., Sechovcová H., Vodička M., Olša Fliegerová K., Dvořáček V., Mrázek J., Pácha J. (2023). Profiling of adrenal corticosteroids in blood and local tissues of mice during chronic stress. Sci. Rep..

[27] Ishack S., Lipner S.R. (2021). Exogenous ochronosis associated with hydroquinone: A systematic review. Int. J. Dermatol..

[28] Searle T., Al-Niaimi F., Ali F.R. (2020). Hydroquinone: Myths and reality. Clin. Exp. Dermatol..

[29] Le L., Sirés-Campos J., Raposo G., Delevoye C., Marks M.S. (2021). Melanosome Biogenesis in the Pigmentation of Mammalian Skin. Integr. Comp. Biol..

[30] Lomenick B., Hao R., Jonai N., Chin R.M., Aghajan M., Warburton S., Wang J., Wu R.P., Gomez F., Loo J.A. (2009). Target identification using drug affinity responsive target stability (DARTS). Proc. Natl. Acad. Sci. USA.

[31] Zeng B., Sun Z., Zhao Q., Liu D., Chen H., Li X., Xing H.R., Wang J. (2021). SEC23A Inhibit Melanoma Metastatic through Secretory PF4 Cooperation with SPARC to Inhibit MAPK Signaling Pathway. Int. J. Biol. Sci..

[32] Ping F., Shang J., Zhou J., Song J., Zhang L. (2012). Activation of neurokinin-1 receptor by substance P inhibits melanogenesis in B16-F10 melanoma cells. Int. J. Biochem. Cell Biol..

[33] Kim D.H., Shin D.W., Lim B.O. (2023). Fermented Aronia melanocarpa Inhibits Melanogenesis through Dual Mechanisms of the PI3K/AKT/GSK-3β and PKA/CREB Pathways. Molecules.

[34] Guo H., Xing Y., Liu Y., Luo Y., Deng F., Yang T., Yang K., Li Y. (2016). Wnt/β-catenin signaling pathway activates melanocyte stem cells in vitro and in vivo. J. Dermatol. Sci..

[35] Brenner M., Hearing V.J. (2008). The protective role of melanin against UV damage in human skin. Photochem. Photobiol..

[36] Cameron S., Donnelly A., Broderick C., Arichi T., Bartsch U., Dazzan P., Elberling J., Godfrey E., Gringras P., Heathcote L.C. (2024). Mind and skin: Exploring the links between inflammation, sleep disturbance and neurocognitive function in patients with atopic dermatitis. Allergy.

[37] Virador V.M., Muller J., Wu X., Abdel-Malek Z.A., Yu Z.-X., Ferrans V.J., Kobayashi N., Wakamatsu K., Ito S., Hammer J.A. (2001). Influence of α-melanocyte-stimulating hormone and of ultraviolet radiation on the transfer of melanosomes to keratinocytes. FASEB J..

[38] Khoriaty R., Hesketh G.G., Bernard A., Weyand A.C., Mellacheruvu D., Zhu G., Hoenerhoff M.J., McGee B., Everett L., Adams E.J. (2018). Functions of the COPII gene paralogs SEC23A and SEC23B are interchangeable in vivo. Proc. Natl. Acad. Sci. USA.

[39] Theos A.C., Berson J.F., Theos S.C., Herman K.E., Harper D.C., Tenza D., Sviderskaya E.V., Lamoreux M.L., Bennett D.C., Raposo G. (2006). Dual Loss of ER Export and Endocytic Signals with Altered Melanosome Morphology in thesilverMutation of Pmel17. Mol. Biol. Cell.

[40] Melville D.B., Montero-Balaguer M., Levic D.S., Bradley K., Smith J.R., Hatzopoulos A.K., Knapik E.W. (2011). The feelgood mutation in zebrafish dysregulates COPII-dependent secretion of select extracellular matrix proteins in skeletal morphogenesis. Dis. Models Mech..

[41] Marshall C.J. (1995). Specificity of receptor tyrosine kinase signaling: Transient versus sustained extracellular signal-regulated kinase activation. Cell.

[42] Murphy L.O., Blenis J. (2006). MAPK signal specificity: The right place at the right time. Trends Biochem. Sci..

[43] Levy C., Khaled M., Fisher D.E. (2006). MITF: Master regulator of melanocyte development and melanoma oncogene. Trends Mol. Med..

[44] Wu M., Hemesath T.J., Takemoto C.M., Horstmann M.A., Wells A.G., Price E.R., Fisher D.Z., Fisher D.E. (2000). c-Kit triggers dual phosphorylations, which couple activation and degradation of the essential melanocyte factor Mi. Genes Dev..

[45] Phan G.H., Fujise K., Imaizumi K., Saito A. (2026). Vesicular Transport Mediated by Endoplasmic Reticulum Stress Sensor BBF2H7 Orchestrates Melanin Production During Melanogenesis. Int. J. Mol. Sci..

[46] Hong C., Zhang Y., Yang L., Xu H., Cheng K., Lv Z., Chen K., Li Y., Wu H. (2024). Epimedin B exhibits pigmentation by increasing tyrosinase family proteins expression, activity, and stability. J. Pharm. Anal..

[47] Granholm N.H., Van Amerongen A.W. (1991). Effects of exogenous MSH on the transformation from phaeo- to eumelanogenesis within C57BL/6J-Ay/a hairbulb melanocytes. J. Investig. Dermatol..

[48] Hong C., Yang L., Zhang Y., Li Y., Wu H. (2022). Epimedium brevicornum Maxim. Extract exhibits pigmentation by melanin biosynthesis and melanosome biogenesis/transfer. Front. Pharmacol..

