# Supplementary material for: Salidroside Selectively Binds to SEC23A and Ameliorates Psychological Stress-Induced Hyperpigmentation
Source: Pharmaceuticals (Basel). 2026 Mar 16;19(3):487. doi: 10.3390/ph19030487 (PMC13029700; doi:10.3390/ph19030487)
Supplement: Supplementary file 1 [file pharmaceuticals-19-00487-s001.zip › Table S1. Nucleotide sequences of PCR primers.pdf]

Gene name	Forward primer (5'->3')	Reverse primer (5'->3')
TYR	CACCTGAGGGACCACTATTACG	CCATCTTTGGAAGGTTCAG GTTG
TYRP1	ATCATCGGCCAAAACGATCAT	GCAGCTAAAATAACAGGT GCGA
DCT	TTCTGCTGGGTTGTCTGGG	CACAGATGTTGGTTGCCTC G
MITF	CAAATGGCAAATACGTTACCCG	CAATGCTCTTGCTTCAGAC TCT
SEC23A	CCTCTGAAGGAGAGACCCGA	TCAAAACTGCACGGCAAGTG
MAPK1	GCATGGTTTGCTCTGCTTATG	GGTTCTTTGACAGTAGGTCTGG
β-Actin	GGGAAATCGTGCGTGAC	AGGCTGGAAAAGAGCCT
