# Supplementary figures and images for: Salidroside Selectively Binds to SEC23A and Ameliorates Psychological Stress-Induced Hyperpigmentation
Source: Pharmaceuticals (Basel). 2026 Mar 16;19(3):487. doi: 10.3390/ph19030487 (PMC13029700; doi:10.3390/ph19030487)

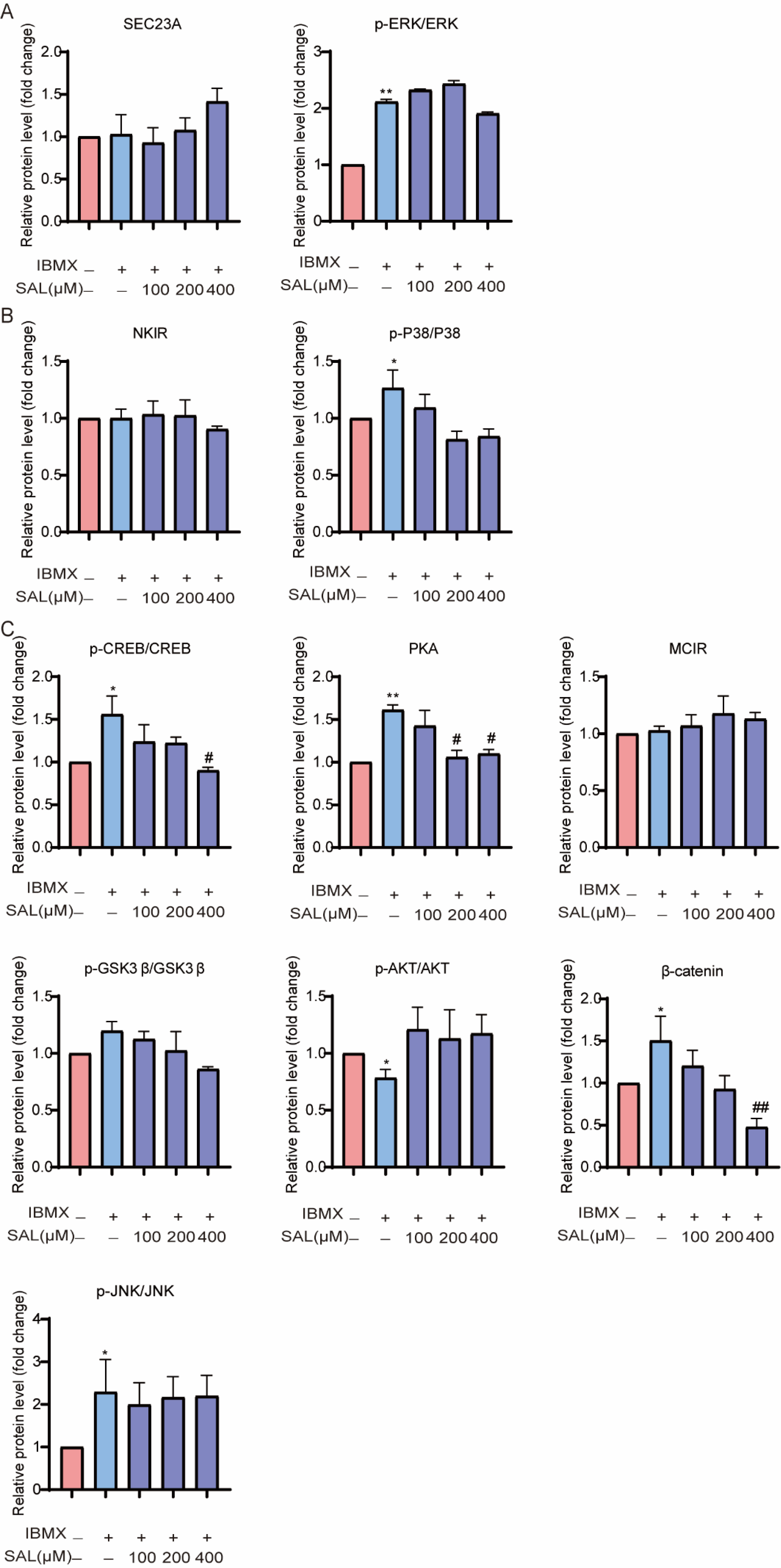

Supplement: Supplementary file 1 [file pharmaceuticals-19-00487-s001.zip › Supplement Figure S5.tif]

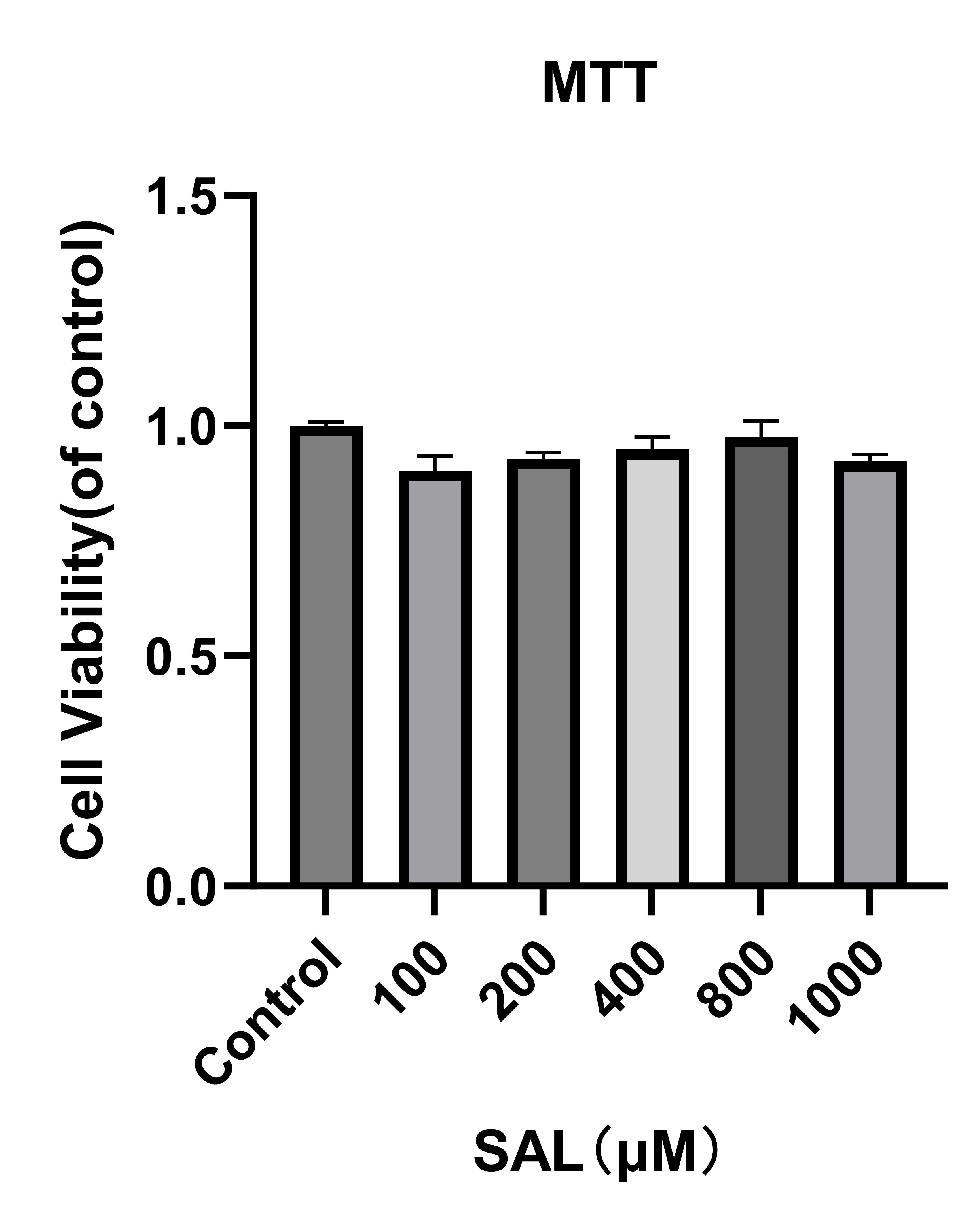

Supplement: Supplementary file 1 [file pharmaceuticals-19-00487-s001.zip › Supplement Figure S1.tif]

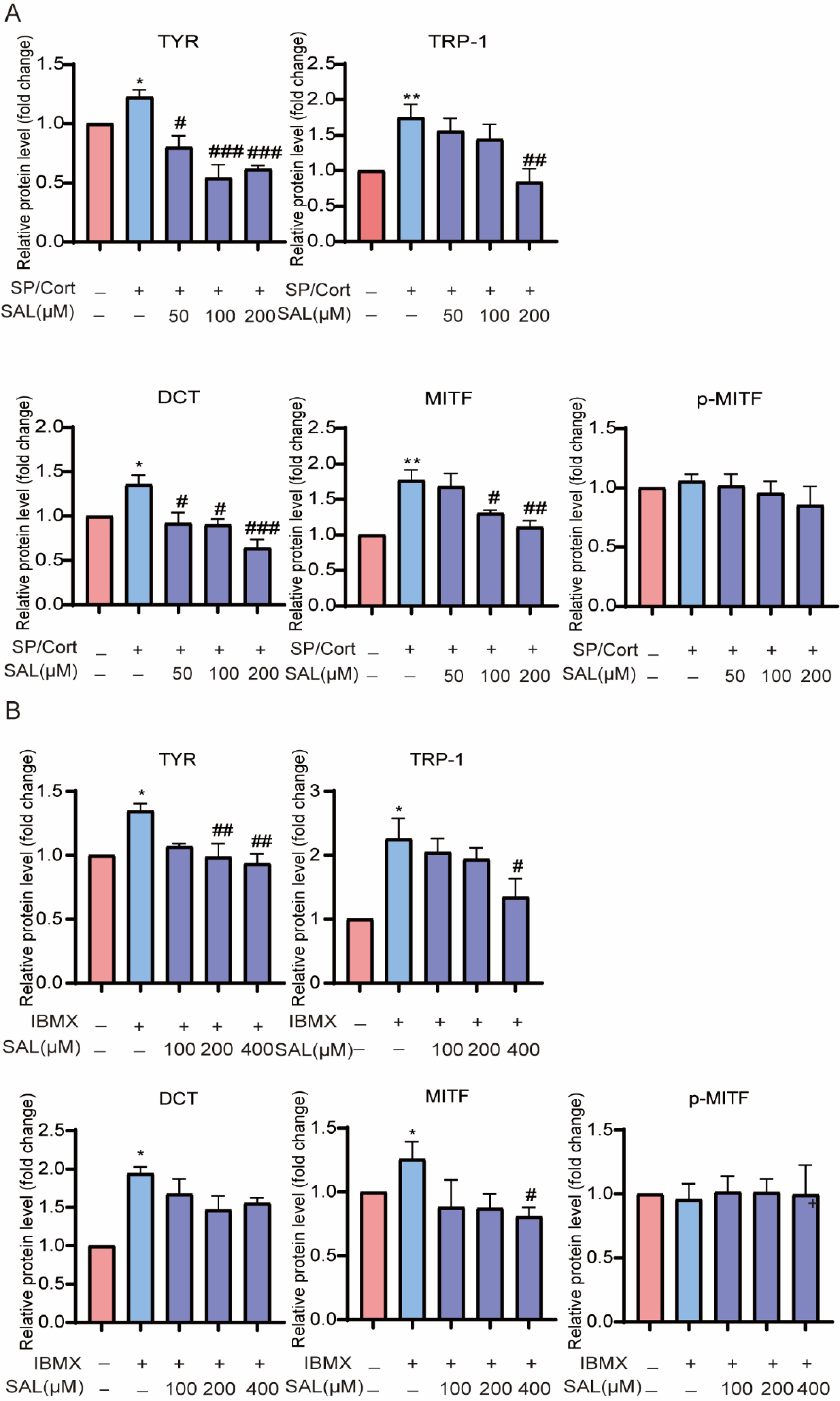

Supplement: Supplementary file 1 [file pharmaceuticals-19-00487-s001.zip › Supplement Figure S2.tif]

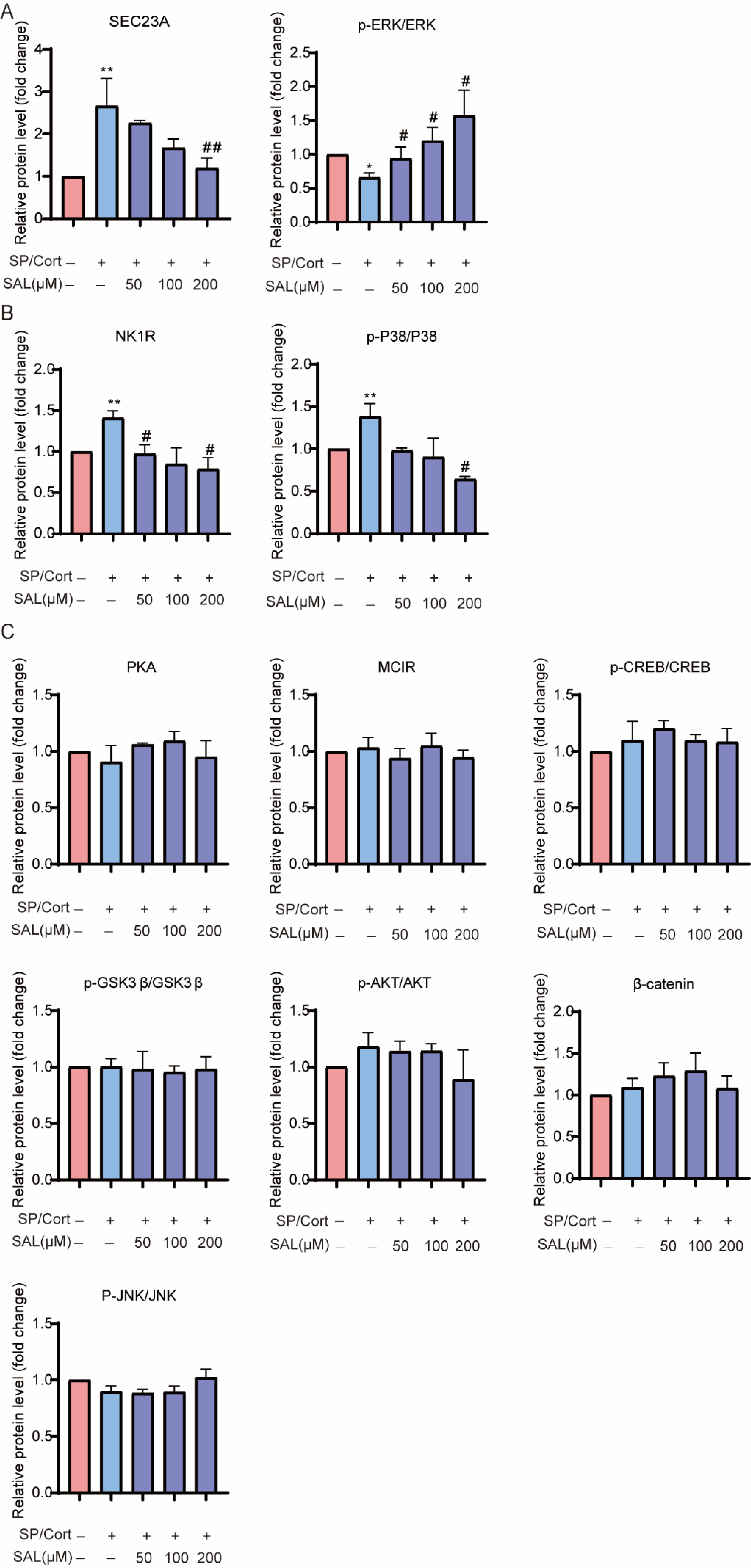

Supplement: Supplementary file 1 [file pharmaceuticals-19-00487-s001.zip › Supplement Figure S4.tif]
